# *Anopheles darlingi* (Diptera: Culicidae) displays increased attractiveness to infected individuals with *Plasmodium vivax* gametocytes

**DOI:** 10.1186/1756-3305-7-251

**Published:** 2014-05-29

**Authors:** Elis PA Batista, Elizangela FM Costa, Alexandre A Silva

**Affiliations:** 1 Departamento de Biologia, Laboratório de Bioecologia de Insetos, Núcleo de Ciência e Tecnologia da Universidade Federal de Rondônia, Campus José Ribeiro Filho, BR 364, Km 9,5, CEP 76801–974, Porto Velho, Rondônia, Brasil; 2 Laboratório de Entomologia, Fundação Oswaldo Cruz – Rondônia, BR 364, Km 1,5, n º 7671, CEP 78912–245, Lagoa, Porto Velho, Rondônia, Brasil

**Keywords:** *Anopheles darlingi*, Malaria, Olfactometer

## Abstract

**Background:**

Most hematophagous insects use host odours as chemical cues. The odour components, some physiological parameters and host attractiveness are affected by several conditions, including infection by parasites, e.g., plasmodia and, therefore, change the epidemiological scenario. This study evaluated the attractiveness of individuals with vivax malaria before, during (7 days) and after treatment (14 days) with specific antimalarial drugs.

**Findings:**

Mosquito attractiveness to vivax-infected patients was assessed using a vertical olfactometer using the foot as a source of body odour. The ratio of *Anopheles darlingi* mosquitoes in the lower chamber of the olfactometer was used to calculate the attractiveness, and patient temperature was measured using a digital thermometer. An increased attractiveness was found only in patients bearing vivax gametocytes during the first experiment (early infection) (P < 0.001). Patients in the first experiment tended to have a higher body temperature, but grouping patients into fever and non-fever resulted in a higher attractiveness only in the fever group of gametocyte carriers, suggesting a synergistic effect of temperature and gametocytes in the host attractiveness to *A. darlingi*.

**Conclusions:**

Gametocyte presence and fever in vivax malaria patients increased short distance host attractiveness to *An. darlingi*.

## Findings

### Background

Few ecological relationships are so intimate such as a parasite and its host. Both antagonists, coexisting over time, evolve an “arms race”, i.e. hosts evolve defense mechanisms, including the immune system to prevent, eliminate or even tolerate parasites that develop new ways to find, explore and manipulate their hosts, that can increase their transmission success.

Vector feeding behavior on hosts can be modified by parasites [1], but parasite infection can result in changes to host physiology that increases the detection rate amongst mosquitoes. Moreover, defensive behavior of vertebrates can be reduced when the hosts are sick, reducing awareness to bites or even inability to respond appropriately to the attack [2]. Changes in the attractiveness and body temperature are also affected during infection by parasites [3].

Despite some evidence regarding the attractiveness of *An. gambiae* to *P. falciparum*-infected individuals [4], the mechanism and compounds mediating attractiveness to infected hosts has not been described so far. Regardless of the mechanisms employed by the parasite in the manipulation of vector and vertebrate host to increased transmission, the result can make a difference on the epidemiology of the disease and, therefore, the understanding of such mechanisms can be used as a strategy to decrease and/or block parasite transmission. The present work investigated the short distance attractiveness response of the main malaria vector in North Brazil, *An. darlingi*, to infected patients with *Plasmodium vivax*, responsible for most of the malaria cases in Brazil.

### Methods

This study was approved by the local Ethics Committee (CEP) of the Research Centre of Tropical Medicine of Rondônia (Cepem - RO), protocol number 091/2009.

The behavioral experiments on *Anopheles darlingi* were performed at the Laboratory of Entomology of the Fundação Oswaldo Cruz (FIOCRUZ-RO) in Porto Velho, Rondonia state, Brazil. Six people completed all the experimental phases and 11 people participated in the first phase. Patients from both sexes, with an age range from 20 to 50 years, diagnosed with vivax malaria with or without gametocytes by blood smear, performed the experiments in a vertical olfactometer [5]. Each experiment was replicated three times using 10 F_1_*An. darlingi* females, which were 5 days old and deprived of a sugar meal for 12 hours. A total of 10 minutes for each experiment included five minutes prior to stimulation with no odour or heat sources and five minutes with odour and heat sources, i.e., patient foot under the lower chamber. Mosquitoes were introduced into the upper chamber of the olfactometer and the result was defined by the sum of mosquitoes present in the lower chamber at the end of all replicates.

Before each experiment, the axillary temperature of the volunteers was measured using a digital thermometer. Each subject that completed all the experimental phases performed the same experiments three times; the first was just after diagnosis (before starting treatment), the second after seven days (during treatment) and the third, 14 days after diagnosis (after treatment). During the last experiment, volunteers had completed treatment and recovered their innate attractiveness, i.e., control.

The mosquito attractiveness of patients that completed all the experimental phases, i.e., before, during and after treatment and the presence of different forms of *P. vivax* were evaluated using Two-Way RM ANOVA and comparisons using Tukey’s test (N = 3). Body temperature of patients throughout the treatment period was evaluated using One Way RM ANOVA (N = 6). Correlation between the body temperature of patients in the first experiment and the number of mosquitoes in the lower chamber of the olfactometer was performed using Pearson correlation. The effect of different forms of *P. vivax* and body temperature, i.e., fever and gametocytes, fever and no gametocytes, no fever and gametocytes and no fever and no gametocytes in mosquito attractiveness was performed using patients within each combination mentioned and evaluated using Two-Way ANOVA and comparisons using Sidak’s test (N = 2) (Prism 6, GraphPad).

### Results

**Figure 1:**
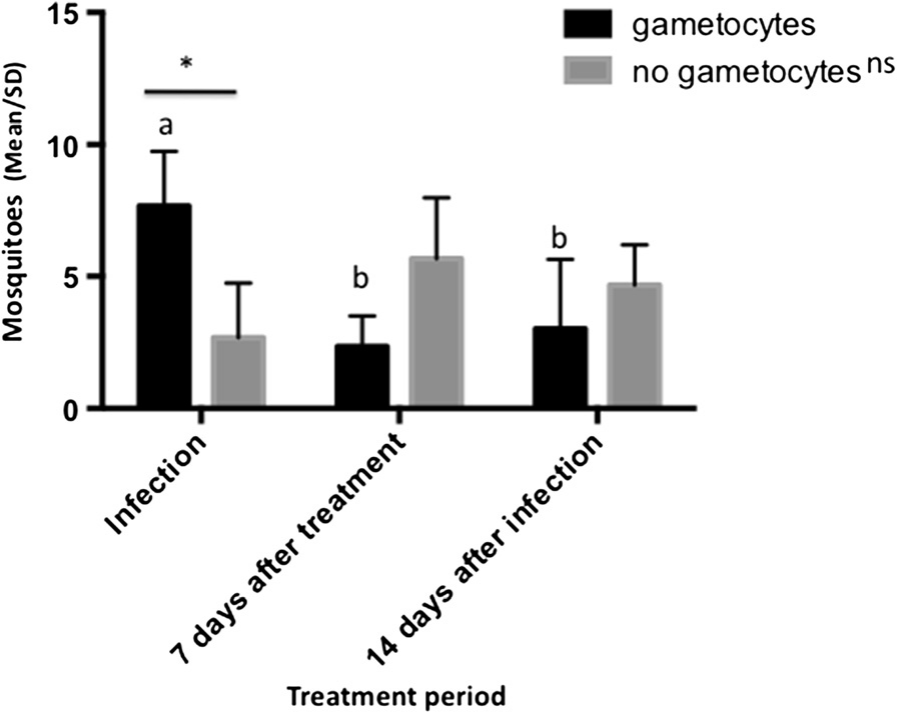
***Anopheles darlingi *attractiveness to vivax malaria patients during different periods throughout treatment.** Two-Way Anova Repeated Measures and Tukey’s test (comparisons). Different letters indicate significant (P < 0.05) differences in mosquito attractiveness to patients bearing gametocytes during the treatment period. NS indicate non-significant (P > 0.05) differences in mosquito attractiveness to patients with no detectable gametocytes during the treatment period. * indicates a significant (P < 0.01) difference in the mosquito attractiveness between patients with detectable gametocytes (gametocytes) (N = 3) and non-detectable gametocytes (no gametocytes) (N = 3) during the infection phase.

**Figure 2:**
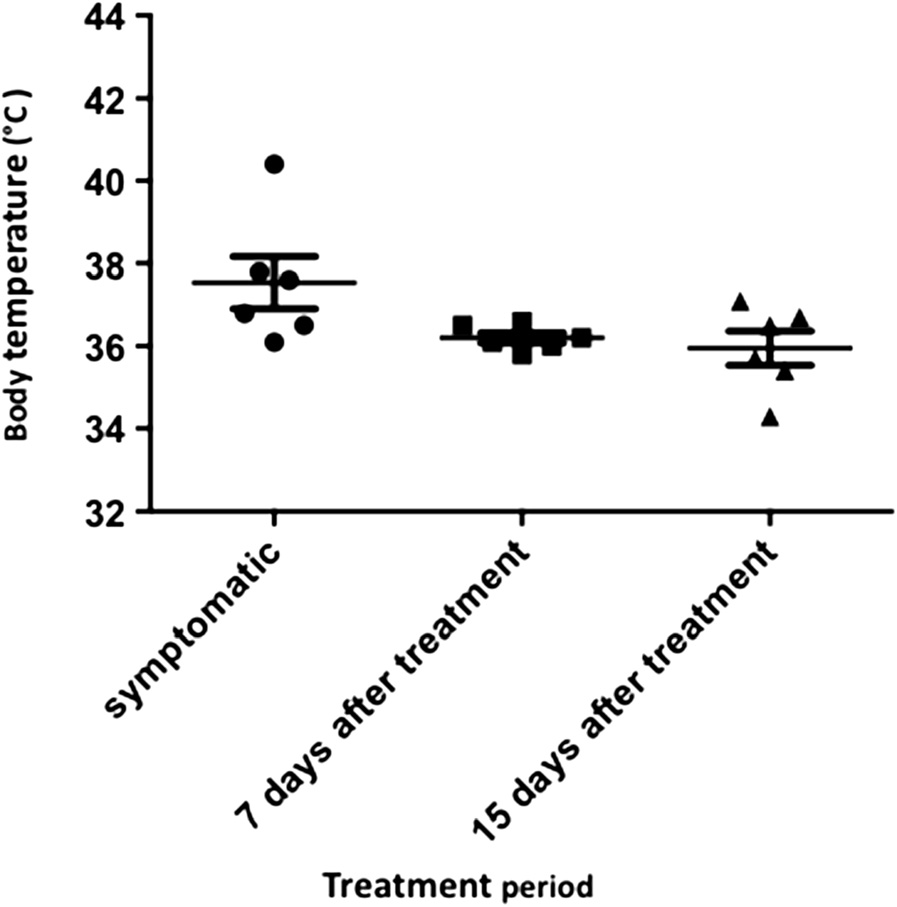
**Body temperature of vivax malaria patients during different periods throughout the treatment.** One Way RM Anova. N = 6.

**Figure 3:**
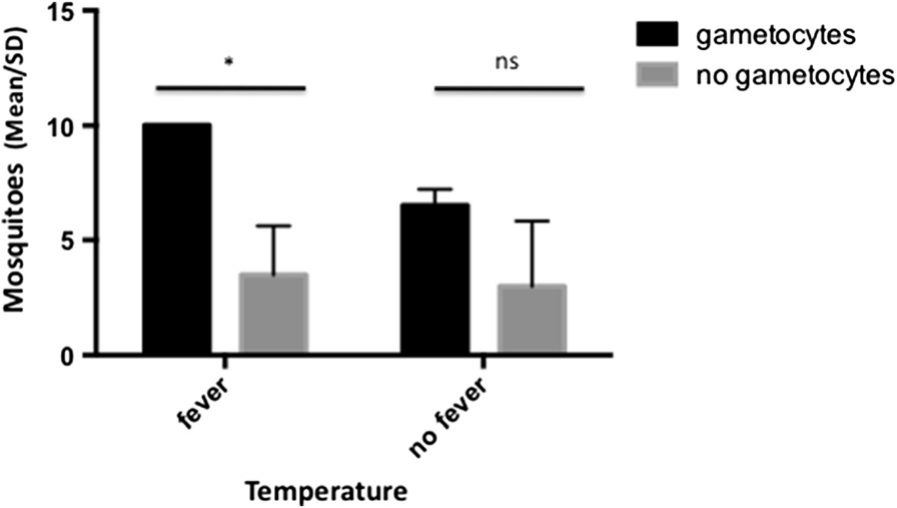
***Anopheles darlingi *attractiveness to symptomatic vivax malaria patients related to body temperature and parasite stages.** Gametocyte carriers were defined as patients with detectable sexual stages of the parasite in blood smears using microscopy. Fever = Individuals with body temperature >37.5°C; No Fever = individuals with body temperature <37.5°C. Two-way ANOVA (fever and gametocytes) and Sidak’s test (comparisons). * indicates significant differences (P < 0.05). NS indicates non-significant differences (P > 0.05). N = 2 for each variable combination (i.e., fever and gametocytes, fever and no gametocytes, no fever and gametocytes and no fever and no gametocytes).

There was a significant interaction between the treatment phase and the presence of gametocytes (F = 15.0, P = 0.0046). The symptomatic patients carrying gametocytes attracted more than twice as many mosquitoes during the infection phase compared with other treatment periods. There were no significant differences in mosquito attractiveness to patients with no detectable gametocytes (P > 0.05) throughout the treatment period. Patients carrying gametocytes attracted 3 times more mosquitoes than patients with no detectable gametocytes only in the infection phase (F = 10.65; P = 0.0056). After 7 and 14 days of treatment, no significant differences in mosquito attractiveness to patients previously infected with different forms of *P. vivax* was detected, but mosquito number tended to be lower in patients previously bearing gametocytes (Figure 1).The average body temperature of patients tended to be higher during the first experiment, but the differences were not significant (F = 4.67, P = 0.053) (Figure 2), and no correlation between body temperature and attractiveness was found (R = 0.38, P = 0.12).However, grouping patients according to their body temperature (fever, i.e., >37.5°C and no fever, i.e., <37.5°C) and the presence of gametocytes (present and absent) in the first experiment indicated that febrile patients bearing gametocytes were more attractive to mosquitoes (F = 15.4, P = 0.017) (Figure 3).

### Discussion

The vertical olfactometer design allows the interaction of physical and chemical cues at a short distance simultaneously during experiments and was successfully performed with *Aedes aegypti*[6]. Despite differences in mosquito response, i.e., higher for *Ae. aegypti*, present data indicate that it is also suitable for experiments with anophelines.

Even with individual innate differences in mosquito attractiveness, the effect of the parasite in the infective stage for the mosquito was significant. In a semi-field condition study conducted in Kenya, Lacroix and colleagues [4] found that children carrying gametocytes attracted twice as many *An. gambiae* in relation to uninfected children and children infected with asexual forms of *Plasmodium*, corroborating the results obtained in the present work for short distance attractiveness. In both studies, increased attractiveness was only observed in experiments prior to treatment with antimalarial drugs. Furthermore, Lacroix and colleagues [4] also reported a decrease in attractiveness after the elimination of the parasite.

Unlike in Lacroix’s study [4], uninfected individuals were not tested in the present study, but experiments were performed with the same individuals during and after treatment, when they had recovered their innate attractiveness and we also found no difference in attractiveness in those previously infected with asexual forms and gametocyte carriers after treatment. Although antimalarial drugs include a known gametocidal drug, i.e., primaquine, gametocyte clearance was not confirmed in the present study.

Although in this study patients were all symptomatic individuals, the increased attractiveness did not result from host behavioral changes, but probably from physiological (odour and temperature) changes, since during the experimental procedures, mosquitoes had no contact with patients. Present results suggest that a specific stage of the parasite, i.e., gametocytes, modify the odour profile of the host, colloquially known as “malaria smell”. Interestingly, increased attractiveness to *Anopheles gambiae* was also reported even in asymptomatic individuals carrying gametocytes [4], prompting their importance as parasite reservoirs, as highlighted by Alves and colleagues [7].

The symptomatic infection probably not only changes the odour profile, but also other important short distance attraction cues such as temperature and humidity. Despite no correlation between temperature and attractiveness in the present work, Eiras & Jepson [6] argued that an increase in temperature in relation to environmental temperature prompted a significant flight response in *Aedes aegypti*. This indicates that physical stimuli are important for orientation at short distances and should be evaluated along with the chemical cues using different approaches for long distance experiments, e.g., taxis assays [8].

Kelly [9] suggested that the change in the innate attractiveness of an infected person results from a diffuse coevolution of parasite, vector and host, indicating a time of increased vulnerability to the bites. Furthermore, increased attractiveness due to host-infected cues seems to be a convergent strategy for host-seeking vectors, e.g. *Lutzomyia longipalpis*[10].

This scenario probably has a significant epidemiological impact on the transmission dynamics of malaria in endemic areas such as Africa [11] and the Brazilian Western Amazon [12] with a high number of asymptomatic carriers of gametocytes. Moreover, Katsuragawa *et al*. [13] studied the dynamics of malaria transmission of an endemic area in Porto Velho, Rondonia and argued that the concentration of malaria cases in some residences might also be a result of a higher frequency of “fever episodes” and therefore increased sweat production by inhabitants of these houses resulting in increased mosquito attraction.

## Conclusions

The use of the vertical olfactometer for studying short distance attractiveness responses to malaria patients was successfully performed with *An. darlingi* and suggests that it is suitable for assays with different mosquito species.

Malaria infection affected the short distance attractiveness of the mosquito *An. darlingi* only in patients carrying gametocytes. Fever was also important to increase host attractiveness.

Since the increased attractiveness for hosts carrying gametocytes may be mediated by olfactory signals, as yet unidentified, studies describing the physiological changes caused by infection are needed to elucidate the factors related to attractiveness.

## Competing interests

The authors declare that they have no competing interests.

## Authors’ contributions

EPAB and AAS conceived and designed the study; EPAB and EFMC performed the experiments and collected the data. EPAB prepared the first draft of the manuscript; AAS provided strategic advice and assisted with editing of the manuscript. All authors read and approved the final version of the manuscript.
